# The Gulf of Mexico: A “Hot Zone” for Neglected Tropical Diseases?

**DOI:** 10.1371/journal.pntd.0003481

**Published:** 2015-02-26

**Authors:** Peter J. Hotez, Maria Elena Bottazzi, Eric Dumonteil, Pierre Buekens

**Affiliations:** 1 Sabin Vaccine Institute and Texas Children’s Hospital Center for Vaccine Development, National School of Tropical Medicine, Baylor College of Medicine, Houston, Texas, United States of America; 2 Department of Biology, Baylor University, Waco, Texas, United States of America; 3 James A. Baker III Institute for Public Policy, Rice University, Houston, Texas, United States of America; 4 Laboratorio de Parasitología, Centro de Investigaciones Regionales "Dr. Hideyo Noguchi", Universidad Autónoma de Yucatán, Mérida, Yucatán, Mexico; 5 School of Public Health and Tropical Medicine, Tulane University, New Orleans, Louisiana, United States of America; Pasteur Institute of Iran, ISLAMIC REPUBLIC OF IRAN

*Subtropical portions of North America that envelop the Gulf of Mexico are emerging as areas that are highly endemic for neglected tropical diseases (NTDs).*

**Fig 1:**
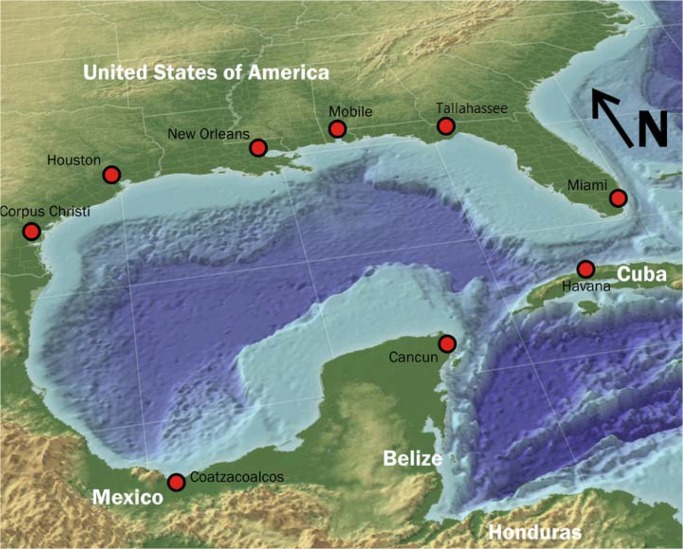
The Gulf of Mexico. http://en.wikipedia.org/wiki/Gulf_of_Mexico#mediaviewer/File:Fixed_gulf_map.png

The ocean basin that comprises the Gulf of Mexico is surrounded by the United States to the north, Mexico to the southwest and Cuba to the southeast. They include both large urban centers such as Houston (6 million), Tampa-St. Petersburg (3 million), Havana (2 million), and Merida, Veracruz, and New Orleans (about 1 million, each), as well as rural populations (Fig. 1).

We previously highlighted the unexpectedly high rates of NTDs emerging in the US Gulf States of Texas, Louisiana, Mississippi, Alabama, and Florida [1]. They include arbovirus infections such as dengue, Chikungunya, and West Nile virus infection; parasitic infections such as Chagas disease, toxocariasis, and trichomoniasis, and selected bacterial infections such as murine typhus and *Vibrio vulnificus* infections [1]. In the case of the US Gulf Coast, the term “emerging” should be used with caution since there is evidence that many of these NTDs are not new to the region. Instead, the Gulf Coast represents one of the poorest regions of the US, and this extreme poverty, together with environmental degradation and a warm subtropical climate, are the key factors that allow NTDs to flourish. Key additional factors might include the absence of investment in active disease surveillance or intervention, lack of access to healthcare, and low recognition or prioritization by local, state, and federal public health agencies.

For similar reasons, NTDs are also widespread along the Gulf Coast states of Mexico—Tamaulipas, Veracruz, Tabasco, Campeche, Yucatan, and Quintana Roo—as well as in Chiapas and Oaxaca on the southern Pacific coast (Table 1). Mosquito-borne diseases are especially prominent. Dengue is now widespread, especially in Yucatan and other coastal and tropical areas [2]. All four dengue serotypes are present, and from 2000 to 2011 the incidence rates increased approximately 8-fold, with peaks in 2002, 2007, and 2009 [2]. West Nile virus has also been detected in circulating birds [3], with the expectation that human infections and disease could also emerge. Vivax malaria accounts for 99 percent of Mexico’s malaria cases and is mostly concentrated in Oaxaca and Chiapas [4,5]. Today, malaria is considered in the “preelimination” phase [5], but the risk for malaria reemergence is considered high, especially during “El Niño” events [4].

**Table 1:** Major NTDs affecting the Gulf of Mexico.

Disease	Current status in United States	Current status in Mexico
**Vector-borne NTDs**		
Vivax malaria	Nonendemic	Endemic
Dengue fever	Emerging	Endemic
West Nile virus infection	Endemic	Emerging
Chagas disease	Endemic	Endemic
Cutaneous leishmaniasis	Emerging	Endemic
Rickettsial infections	Endemic	Endemic
**Helminthic NTDs**		
Soil-transmitted helminth infections	Not determined	Endemic
Cysticercosis	Endemic	Endemic
Toxocariasis	Endemic	Endemic
Fascioliasis	Nonendemic	Endemic
**Other NTDs**		
Intestinal protozoan infections	Endemic	Endemic
Leptospirosis	Sporadic or emerging	Emerging

Among the other vector-borne parasitic infections, most of the cases of cutaneous leishmaniasis (CL) are found in the Tabasco state, where it has been linked to the cacao industry and plantations [6]. CL is also found in Veracruz, as well as in Yucatan on the Gulf Coast [7–9]. While *Leishmania mexicana* is the major cause of human CL in Mexico, *Leishmania braziliensis* and *Leishmania infantum* are also found, and all three species are thought to be circulating in dogs in Yucatan and elsewhere along the coastal region [7], with *L. mexicana*, the cause of “chiclero’s ulcer,” a major etiologic of human CL [8]. Visceral leishmaniasis has also been reported [10]. Chagas disease (American trypanosomiasis caused by *Trypanosoma cruzi*) remains an important NTD in Mexico, with more than 1 million cases [11]. The highest prevalence of Chagas disease occurs in the southern states, including Oaxaca and Chiapas [12]. In Yucatan and elsewhere on the Gulf Coast, the prevalence is approximately one percent, although some investigators report much higher prevalence rates [12], mostly associated with transmission from *Triatoma dimidiata* kissing bugs [13,14]. The presence of these triatomine vectors in turn has been linked to the presence of dogs and chickens and several key bug refuges including rock piles and vegetation at the village peripheries [15]. The rate of *T. cruzi* vector-borne transmission has been estimated at approximately one human infection per 900–4,000 contacts with triatomines [16]. Among rural Mayan communities on the Yucatan peninsula, *T. cruzi* infection has been identified among children and their mothers, suggesting both maternal-to-child transmission and vector-borne transmission. The same study showed that *T. cruzi* infection is associated with a history of poor reproductive outcomes [17]. Human rickettsialpox is an important vector-borne bacterial infection in southeastern Mexico [13,18]; *Rickettsia felis* has emerged as a flea-borne bacterial infection [19].

Taeniasis or cystcercosis, toxocariasis, and leptospirosis are key zoonotic NTDs [20–22]. Intestinal helminth and protozoan infections are believed to be widespread, especially in southern Mexico [23].

There are important opportunities for international cooperation between the governments of the US and Mexico and both public sector and private scientific institutions located in the Gulf of Mexico region in order to control or eliminate selected NTDs. They include active disease surveillance to assess the rural versus urban distribution of the major NTDs, deeper knowledge about the modes of transmission, and the role of marginalized (including indigenous) populations affected by these conditions. Shared vector control efforts for *Aedes* mosquitoes linked to dengue and chikungunya, and for *Culex* mosquitoes that transmit West Nile virus infection, represent key interventions. Vector control is also a cornerstone for triatomine vector reductions to combat Chagas disease in Yucatan and elsewhere in southeastern Mexico [14,15]; it may also help to control some rickettsial infections. Deworming and other intestinal parasite control measures are still required. According to the World Health Organization, approximately 10 million preschool- and school-aged children require regular and periodic deworming in Mexico [23]. Joint efforts are needed for some of the key zoonotic NTDs in the region including taeniaisis or cysticercosis, toxocariasis, fascioliasis, and leptospirosis. Shared efforts for research and development to produce new or improved diagnostics, drugs, and vaccines are also needed.

Towards these goals, the two schools of tropical medicine located on the US Gulf Coast—the National School of Tropical Medicine at Baylor College of Medicine and the Tulane University School of Public Health and Tropical Medicine have embarked on collaborative efforts with the “Dr. Hideyo Noguchi” Regional Research Center of the Autonomous University of Yucatan (UADY). A major focus of this joint effort is Chagas disease [24], both in the areas of vector control and efforts to interrupt maternal-to-child transmission [13–17,25] and in the development of new control tools, including a therapeutic vaccine, either as a stand-alone product or one used in concert with antiparasitic drugs [26]. Research on Chagas Disease among mothers and children was funded by the Thrasher Research Fund [17] and the National Institute of Allergy and Infectious Diseases (NIAID) [25]. Vaccine development is being led and sponsored by the Carlos Slim Health Institute and involves a consortium of Mexican institutions together with the National School of Tropical Medicine, the Sabin Vaccine Institute, and the Southwest Electronic Energy Medical Research Institute, based in Houston, Texas [26]. Joint cooperation for vaccine development between nations—also known as vaccine diplomacy—has been proposed as a path forward to develop NTD vaccines and other life-saving products [27], including potential interactions between the US and other Latin American nations to specifically target NTDs in the Americas [28]. Beyond such international scientific collaborations in the way of development of new approaches to control and elimination and specific control tools, it would be attractive to determine how such an academic consortium might also help to promote primary healthcare and health systems in the region.

As these pathogens and diseases do not adhere to territorial boundaries, international collaboration will be essential to combat these cross-border diseases. A consortium of Gulf of Mexico biomedical institutions would be a means to advance disease control and elimination efforts in the region. We are eager to launch such a “Trop-G” (Medicina Tropical en el Golfo de México or Tropical Medicine in the Gulf of Mexico) initiative.
